# Supplementary material for: Relationships between Obesity, Nutrient Supply and Primary Open Angle Glaucoma in Koreans
Source: Nutrients. 2020 Mar 24;12(3):878. doi: 10.3390/nu12030878 (PMC7146578; doi:10.3390/nu12030878)
Supplement: Supplementary file 1 [file nutrients-12-00878-s001.pdf]

Supplementary Materials

Table S1. Difference of diet intake between glaucoma and non-glaucoma group in non-obese female group. (Bmi<25)

Diet	Glaucoma (n=99)	Non-glaucoma (n=2,699)	p value		
			Model 1*	Model 2†	Model 3‡
Intake of food (g)	1171.59(77.78)	1337.35(17.88)	0.139	0.025	0.022
Energy (kcal)	1576.95(70.40)	1743.32 (18.48)	0.118	0.037	0.032
Protein intake (g)	53.09(3.23)	63.76(0.99)	0.033	0.011	0.008
Fat intake (g)	28.02(2.53)	37.10(0.69)	0.129	0.008	0.006
Carbohydrate intake (g)	276.22(12.27)	288.36(2.94)	0.159	0.109	0.098
Crude fiber intake (g)	6.33(0.53)	6.82(0.13)	0.144	0.139	0.106
Ash intake (g)	16.73(1.06)	18.12(0.26)	0.314	0.262	0.229
Calcium intake (mg)	426.02(41.29)	476.68(7.76)	0.261	0.069	0.061
Phosphorus intake (mg)	934.07(56.15)	1054.81(12.56)	0.068	0.013	0.010
Iron intake (mg)	12.52(0.99)	13.65(0.43)	0.146	0.193	0.157
Sodium intake (mg)	4250.59(308.60)	4357.62(74.70)	0.963	0.900	0.862
Potassium intake (mg)	2400.54(147.56)	2813.89(39.06)	0.005	0.002	0.002
Vitamin A intake (μg RE)	711.54(88.62)	756.67(19.12)	0.628	0.690	0.637
B-carotene intake (μg)	3506.67(492.33)	3826.80(102.36)	0.399	0.520	0.480
Retinol intake (μg)	105.25(26.09)	106.15(5.60)	0.556	0.934	0.967
Thiamin intake (mg)	1.01(0.06)	1.19(0.02)	0.053	0.006	0.004
Riboflavin intake (mg)	0.99(0.08)	1.13(0.02)	0.417	0.197	0.172
Niacin intake (mg)	12.45(0.73)	14.97(0.22)	0.012	0.004	0.003
Vitamin C intake (mg)	88.72(10.08)	110.02(2.68)	0.046	0.001	<0.001

Data are presented as mean (standard error).

*Model 1: adjusted for age

†Model 2: adjusted for age, diabetes, hypertension, high-density lipoprotein cholesterol, intraocular pressure.

‡Model 3: adjusted for age, diabetes, hypertension, high-density lipoprotein cholesterol, body mass index, intraocular pressure, smoking, drinking alcohol.

Table S2. Difference of diet intake between glaucoma and non-glaucoma group in non-obese males. (Bmi<25)

Diet	Glaucoma (n=106)	Non-glaucoma (n=1,479)	p value		
			Model 1*	Model 2†	Model 3‡
Intake of food (g)	1702.36 (102.34)	1837.64(32.26)	0.480	0.559	0.568
Energy (kcal)	2326.67(99.25)	2517.37(31.46)	0.271	0.249	0.248
Protein intake (g)	80.36(3.72)	93.77(1.67)	0.043	0.019	0.021
Fat intake (g)	45.82(3.91)	55.39(1.21)	0.426	0.226	0.236
Carbohydrate intake (g)	363.57(13.89)	372.87(4.30)	0.590	0.653	0.693
Crude fiber intake (g)	8.43(0.48)	8.02(0.15)	0.687	0.735	0.671
Ash intake (g)	24.36(1.25)	24.51(0.39)	0.880	0.842	0.844
Calcium intake (mg)	636.10(45.40)	588.48(10.79)	0.248	0.145	0.146
Phosphorus intake (mg)	1375.86(51.92)	1455.41(19.10)	0.450	0.407	0.436
Iron intake (mg)	17.14(1.15)	17.59(0.31)	0.617	0.907	0.931
Sodium intake (mg)	5969.61(380.99)	6171.19(114.39)	0.680	0.556	0.530
Potassium intake (mg)	3418.04(152.70)	3596.19(51.29)	0.442	0.441	0.483
Vitamin A intake (μg RE)	874.03(64.46)	1014.45(40.74)	0.169	0.446	0.422
B-carotene intake (μg)	4401.72(348.79)	5214.96(240.35)	0.096	0.322	0.301
Retinol intake (μg)	145.67(31.32)	132.59(5.83)	0.278	0.316	0.309
Thiamin intake (mg)	1.49(0.09)	1.70(0.03)	0.171	0.069	0.082
Riboflavin intake (mg)	1.45(0.11)	1.52(0.03)	0.822	0.810	0.793
Niacin intake (mg)	19.55(1.09)	21.96(0.39)	0.178	0.163	0.184
Vitamin C intake (mg)	110.72(7.77)	116.74(2.84)	0.617	0.924	0.955

Data are presented as mean (standard error).

*Model 1: adjusted for age

†Model 2: adjusted for age, diabetes, hypertension, high-density lipoprotein cholesterol, intraocular pressure.

‡Model 3: adjusted for age, diabetes, hypertension, high-density lipoprotein cholesterol, body mass index, intraocular pressure, smoking, drinking alcohol.
